# Vascular Tissue Specific miRNA Profiles Reveal Novel Correlations with Risk Factors in Coronary Artery Disease

**DOI:** 10.3390/biom11111683

**Published:** 2021-11-12

**Authors:** Katrīna D. Neiburga, Baiba Vilne, Sabine Bauer, Dario Bongiovanni, Tilman Ziegler, Mark Lachmann, Simon Wengert, Johann S. Hawe, Ulrich Güldener, Annie M. Westerlund, Ling Li, Shichao Pang, Chuhua Yang, Kathrin Saar, Norbert Huebner, Lars Maegdefessel, DigiMed Bayern Consortium, Rüdiger Lange, Markus Krane, Heribert Schunkert, Moritz von Scheidt

**Affiliations:** 1 Bioinformatics Lab, Riga Stradiņš University, LV-1007 Riga, Latvia; KatrinaDaila.Neiburga@rsu.lv; 2 SIA Net-OMICS, LV-1011 Riga, Latvia; 3 German Heart Centre Munich, Department of Cardiology, Technical University Munich, 80636 Munich, Germany; s.bauer@tum.de (S.B.); johann.hawe@tum.de (J.S.H.); u.gueldener@tum.de (U.G.); annie.westerlund@tum.de (A.M.W.); lingjoyo.li@tum.de (L.L.); shichao.pang@tum.de (S.P.); chuhua.yang@tum.de (C.Y.); 4 DZHK (German Centre for Cardiovascular Research), Partner Site Munich Heart Alliance, 80802 Munich, Germany; bongiovanni@tum.de (D.B.); lars.maegdefessel@tum.de (L.M.); lange@dhm.mhn.de (R.L.); krane@dhm.mhn.de (M.K.); 5 Department of Internal Medicine I, School of Medicine, University Hospital Rechts der Isar, Technical University of Munich, 81675 Munich, Germany; tilman.ziegler@tum.de (T.Z.); mark.lachmann@uni-duesseldorf.de (M.L.); 6 Helmholtz Pioneer Campus, Helmholtz Zentrum München, 85764 Neuherberg, Germany; simon.wengert@helmholtz-muenchen.de; 7 Institute of Computational Biology, Helmholtz Zentrum München, 85764 Munich, Germany; 8 Max-Delbrück-Center for Molecular Medicine in the Helmholtz Association (MDC), 13125 Berlin, Germany; ksaar@mdc-berlin.de (K.S.); nhuebner@mdc-berlin.de (N.H.); 9 DZHK (German Centre for Cardiovascular Research), Partner Site Berlin, 10785 Berlin, Germany; 10 Charité-Universitätsmedizin, 10117 Berlin, Germany; 11 Department of Vascular and Endovascular Surgery, Klinikum Rechts der Isar, Technical University Munich, 81675 Munich, Germany; 12 German Heart Centre Munich, Department of Cardiac Surgery, Technical University Munich, 80636 Munich, Germany; 13 Division of Cardiac Surgery, Yale University School of Medicine, New Haven, CT 06510, USA

**Keywords:** arteria mammaria interna, biomarker, biosensor, coronary artery disease, coronary artery bypass grafting, cardiovascular disease, local therapy, micro RNA, personalized medicine

## Abstract

Cardiovascular disease (CVD) is the leading cause of morbidity and mortality worldwide. Non-coding RNAs have already been linked to CVD development and progression. While microRNAs (miRs) have been well studied in blood samples, there is little data on tissue-specific miRs in cardiovascular relevant tissues and their relation to cardiovascular risk factors. Tissue-specific miRs derived from *Arteria mammaria interna* (IMA) from 192 coronary artery disease (CAD) patients undergoing coronary artery bypass grafting (CABG) were analyzed. The aims of the study were 1) to establish a reference atlas which can be utilized for identification of novel diagnostic biomarkers and potential therapeutic targets, and 2) to relate these miRs to cardiovascular risk factors. Overall, 393 individual miRs showed sufficient expression levels and passed quality control for further analysis. We identified 17 miRs–miR-10b-3p, miR-10-5p, miR-17-3p, miR-21-5p, miR-151a-5p, miR-181a-5p, miR-185-5p, miR-194-5p, miR-199a-3p, miR-199b-3p, miR-212-3p, miR-363-3p, miR-548d-5p, miR-744-5p, miR-3117-3p, miR-5683 and miR-5701–significantly correlated with cardiovascular risk factors (correlation coefficient >0.2 in both directions, *p*-value (*p* < 0.006, false discovery rate (FDR) <0.05). Of particular interest, miR-5701 was positively correlated with hypertension, hypercholesterolemia, and diabetes. In addition, we found that miR-629-5p and miR-98-5p were significantly correlated with acute myocardial infarction. We provide a first atlas of miR profiles in IMA samples from CAD patients. In perspective, these miRs might play an important role in improved risk assessment, mechanistic disease understanding and local therapy of CAD.

## 1. Introduction

Cardiovascular diseases (CVD) including Coronary Artery Disease (CAD), stroke and peripheral artery disease are the main leading cause of morbidity and mortality worldwide, posing a huge socio-economic burden to the society and health systems [1,2]. CAD is a multi-factorial disease with complex etiology, considered to be driven by both genetic and environmental factors [3,4,5]. Environmental factors can be divided into non-lifestyle-associated factors (e.g., healthcare system, air pollution, socio-cultural living environment) and lifestyle-associated factors (classic risk factors like smoking or high-fat diet). Over the last 15 years, several large-scale genome-wide association studies (GWAS) have successfully identified >300 genetic loci robustly associated with CAD risk [6,7,8,9,10,11,12,13,14,15,16]. Several risk loci have been linked to candidate genes involved in gene regulation via transcription factors. We recently identified that the MAF bZIP transcription factor F (*MAFF*) downregulates low-density lipoprotein receptor (*LDLR*) expression under inflammatory conditions via heterodimerization with BTB domain and CNC homolog 1 (*BACH1*) and binding at the maf-recognition element in the promoter of *LDLR* linking two major causes of CAD development–cholesterol metabolism and inflammation [17]. Currently, the main challenge is the prioritization of putative functional variants [18,19,20,21,22,23,24,25], as >90% of CAD risk loci are located outside the protein-coding regions [18,19], and seem to affect transcription (promoters, enhancers) and non-coding RNAs, including miRs [26]. Genetic variants can affect the functionality of miRs at all levels, including transcription (enhancer, promoter SNVs), processing and maturation, as well as their stability and target specificity [26], or their reciprocal interactions with long non-coding RNAs (lncRNAs) [27]. Moreover, these perturbations would have a system-wide effect, as ~30% of all human genes might be regulated by miRs [28], usually operating on the whole pathway level [29]. Indeed, miRs are increasingly recognized as mediators of cardiovascular disorders [29,30,31] and several studies have investigated their diagnostic value as biomarkers in plasma samples in relation to CAD risk factors [28,32]. 

Coronary artery disease (CAD) also known as ischemic heart disease is the progressive pathological development of atherosclerosis in coronary arteries [33]. Atherosclerosis is a chronic inflammatory process of arteries in response to continuous biological effects of certain risk factors [34]. Atheroprogression contains increasing adhesive properties of the arterial wall as a response to biochemical stimuli, aggregation and maturation of leucocytes to foam cells, smooth muscle cell migration, lipoprotein binding and oxidation which propagates inflammatory response. Furthermore, growth of the lesion can involve calcification, formation of necrotic core, development of arterial stenosis and eventually development of thrombosis and plaque rupture. This has been previously described in detail [35,36]. According to the Global Burden of Diseases, Injuries, and Risk Factors Study (GBD), in 2019 CAD was the second of the leading causes of mortality worldwide among all ages, and the leading cause of mortality in the group above 50 [37]. Overall, it is responsible for about 30% of all deaths in ages 35 and older [38].

Micro RNAs (miRs) are 22–25 bp long, endogenous non-protein-coding RNA molecules with huge regulatory impact. Usually, miRs are transcribed from DNA into primary miRs (pri-miRNAs) and processed by Dosha and Dicer enzymes into precursor miRs (pre-miRNAs) undergoing some cleaving events to form mature miRs. The most common canonical effector mechanism of miR is suppression of target mRNA expression by interacting with 3′ untranslated region (3′UTR) [39]. However, non-canonical biogenesis pathways producing functional miRs have been reported, as well as additional variety of interactions by miRs with other structures, including promoter sequences, coding sequences and 5′ untranslated regions (5′UTR) of mRNA [40,41]. Furthermore, other mechanisms of miR regulatory influence have also been reported, including translational activation and transcriptional and post transcriptional gene regulation [39]. In addition to varied intracellular roles miRs are also secreted in extracellular fluids cell-cell communication [42,43]. Regulatory patterns exhibited by miR towards target mRNA are not straightforward either-multiple miRs can target the same mRNA, and at the same time it is possible for one miR to target multiple mRNAs, creating complex regulatory network interactions [44,45]. The focus of this study was on tissue specific miR patterns and their implications on coronary artery disease. 

## 2. Materials and Methods

The study cohort consists of 192 individuals and was obtained from the German Heart Centre Munich based on a single-centre, prospective, observational trial studying molecular signatures in CAD patients undergoing CABG surgery between 02/2019 and 10/2020 (273/18 s–registered 06/2018). Clinical information, blood and tissue samples were obtained from all patients routinely. Patients with acute ST-elevation MI (STEMI) or prior STEMI based on ICD-10 codes (I21.0, I21.1, I21.2, I21.3 and I21.9) were excluded. Only individuals with available *Arteria mammaria interna* (IMA) samples were considered. Tissue samples were collected in Nunc^®^ cryo-tubes (Thermo Scientific, Schwerte, Germany) after surgical acquisition and immediately snap frozen in liquid nitrogen without further stabilization media. All samples are stored in liquid nitrogen in an established clinical biobank system at the German Heart Centre Munich until further processing. The observational study was conducted in accordance with the provisions of the Declaration of Helsinki and the International Conference on Harmonization Good Clinical Practice guidelines. The protocol was approved by an independent ethics committee at the Technical University Munich (Project 5943/13) and all patients provided written informed consent. Data used in this study are available in persistent repositories at the German Heart Centre Munich and can be requested from qualified researchers.

Clinical data was assessed after admission based on the electronic hospital information system and is available in persistent repositories at the German Heart Centre Munich. Focus was on basic information (e.g., sex, weight, BMI), relevant clinical information and established CVD risk factors following recent guidelines of the European Society of Cardiology (ESC) on cardiovascular disease prevention in clinical practice [46]. The data comprised in detail obesity (yes or no); diabetes (categories ‘no’, ‘borderline diabetes; dietary treatment’, ‘yes, insulin treatment’, ‘yes, oral antidiabetics treatment’, ‘yes, insulin + oral antidiabetics treatment’, ‘yes, untreated’); hyperlipidemia (categories ‘no’, ‘yes, treated’, ‘yes, untreated’, ‘yes, no treatment info’); hypertension (categories ‘no’, ‘yes, treated’, ‘yes, untreated’, ‘yes, no treatment info’); smoking status (categories ‘never’, ‘in past’, ‘currently’); family disposition (yes or no); renal disorder (categories ‘none’ ‘compensated’, ‘dialysis’, ‘kidney transplant with dialysis’, ‘kidney transplant without dialysis’); chronic lung disease or chronic obstructive pulmonary disease (COPD; yes or no). 

RNA extraction. In this study, *n* = 192 snap frozen IMA samples were used for further semi-automated miR extraction using Maxwell RSC miRNA Tissue Kit (AS1460) based on a Maxwell RSC 48 system (Promega) at the German Heart Centre Munich. After removal of surgical vascular clamps and perivascular tissue 20–50 mg per IMA sample were processed. The in house established protocol contained mechanical (Tissue Lyser II, Qiagen, Hilden, Germany) and chemical (Lysis buffer with thioglycerol) as well as enzymatic digestion of the frozen tissue. After mechanical homogenization the samples were lyzed and digested in a proteinase K containing buffer followed by semiautomated RNA extraction according to the company’s protocol, and eluted in 65 µL nuclease free water concentration was measured spectrometrically (Tecan) and miR samples were subsequently stored at −80 °C.

miRNA sequencing and data pre-processing. Concentration and integrity of the miR was measured with Bioanalyzer 2100 and the Agilent Small RNA Kit (Agilent Technologies, Waldbronn, Germany). Thereafter, 2 μg of total RNA was used to prepare miR libraries with the TruSeq Small RNA Library Prep Kit (Illumina, San Diego, CA, USA) and sequenced on the HiSeq 4000 System at a 50 bp read length and a depth of ≥40 million reads. Quality control was done using FastQC version 0.11.9 [47] and Fastp version 0.20.0. with overrepresentation analysis enabled [48]. Adapter trimming was done using Fastp version 0.20.0. with the following settings: --adapter_sequence = TGGAATTCTCGGGTGCCAAGG -5 -3 Adapter sequence was specified based on Illumina reference for TruSeq Small RNA library preparation kit. Options -5 and −3 enable sliding window cutting from both ends by mean quality score evaluation with default settings: mean quality threshold 20 and sliding window size 4. Finally, the outcome of pre-processing steps was merged and visualized with MultiQC version 1.9 [49]. Sequence alignment to the refSeq and miR identification was performed using human genome version GRCh38.p13 primary assembly fasta file obtained from ensembl.org for genome reference. Mature miR sequences were downloaded from miRBase (http://www.mirbase.org/ release 22.1 accessed on 1 July 2021). Mapping and miR identification were done using miRDeep2 version 0.1.3. [50]. Sequences for human (*Homo sapiens*), and for additional related miR identification, if needed, including the house mouse (*Mus musculus*), Bornean orangutan (*Pongo pygmaeus*), western gorilla (*Gorilla gorilla*), rhesus macaque (*Macaca mulatta*) and chimpanzee (*Pan troglodytes*), were extracted from mature miR fasta files. Related species were chosen based on closeness in taxonomy to the human and the number of annotated miRs in miRBase. Besides related species miR information, default options were used both for the mapper.pl script mapping to the reference genome and the miRDeep2.pl script for identifying and quantifying miRs. After mapping to miRBase, to avoid overlapping due to historical nomenclature changes, names of all the miRs were standardized to miR-X-M-Np (in some cases let-X-M-Np), where X–sequential unique ID number of the miR; M–location identifier of identical miR from different locations (from older nomenclature); Np–arm of the pri-miRNA from which the mature sequence is derived (3p for 3′ arm and 5p for 5′ arm). Afterwards to avoid unnecessary inflating the number of multiple comparisons, the expression levels of the same miRNAs coming from differently located precursors were combined and all names standardized to miR-X-Np (in some cases let-X-Np) according to aforementioned explanation.

Data analysis and integration with clinical data. After removing features with consistently low counts (median < 10), the raw count data was normalized using the trimmed mean of M-values (TMM) normalization method [51], as implemented in the edgeR package [52]. Continuous clinical variables were standardized to z-scores prior to the correlation analysis and samples with no clinical measurement values in the respective categories were excluded prior to the analyses. For continuous clinical variables, the Kendall Correlation Coefficient (τ) was calculated, using the base package stats of the R statistical software (version 4.0.5). For discrete clinical variables, i.e., those that had only two categories, the Point-Biserial correlation (rpb) was calculated as the Pearson’s Bivariate Correlation Coefficient, also using ‘stats’. Benjamini-Hochberg multiple testing correction (BH or the false discovery rate (FDR)) was used for the adjustment of *p*-values [53]. Data were analyzed using R statistical software version 4.0.3. In particular, the packages tidyverse and janitor were used for general data wrangling and cleaning, whereas the package ggplot2 was used for data visualization.

## 3. Results

### 3.1. Study Workflow

**Figure 1:**
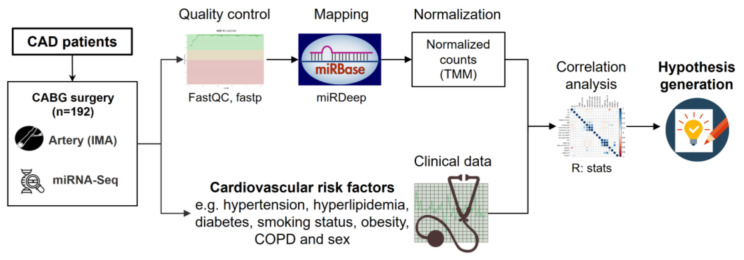
Provided is the workflow of the study including study cohort, miR sequencing, bio-informatics methods, relevant clinical information and analysis plan.

The workflow of the study is summarized in Figure 1. IMA samples of 192 individuals undergoing CABG were collected for further investigation. After quality control, mapping and normalization of miR sequencing data miR profiles were correlated to assessed cardiovascular risk factors.

### 3.2. Study Cohort

A total of 192 individuals with angiographically assessed CAD were enrolled. Four individuals were excluded from the final analysis for either not passing quality control assessment (*n* = 1) or lacking relevant clinical variables of interest (*n* = 3). Baseline characteristics of the final study cohort (*n* = 188) are summarized in Table 1. 

Women (*n* = 28) were underrepresented compared to men (*n* = 160) in this contemporary bypass cohort (14.9% vs. 85.1%; *p* < 0.001). Baseline characteristics between groups were balanced showing no significant differences with regard to age, BMI, cardiovascular risk factors, clinical presentation, medical history, reduced left-ventricular ejection fraction (LV-EF) and preoperative medication. The proportion of individuals with coronary 3-vessel disease (89.4% vs. 67.9%, *p* = 0.006) and hemoglobin values (14.0% vs. 12.0%, *p* = 0.04) were higher in male individuals. 

### 3.3. miRNAs in IMA Samples—A Novel Atlas

miRNA quality assessment showed excellent sequencing quality for 191 IMA samples. Quality (phred) scores per base and sequence reached the accepted ‘good quality’ threshold of >25, and lowest phred score for first five bp of reads was >30 for all samples. The quality scores per sequence did not show outlier sequences with lower quality (Figure S1). Adapter content of sequences are provided before and after trimming. Based on Illumina TruSeq Small RNA adapter sequence, adapter content dropped below 2% after trimming. (Figure S2). In total, 393 individual miRs were identified reaching a median expression level of at least ten reads across all samples. These results will serve as a future compendium or atlas of miRs in IMA samples of CAD patients. The complete list of miRs including expression values across the study cohort of 191 individuals is provided in Table S1. 

### 3.4. miRNAs and Cardiovascular Risk Factors

All 393 miRs were correlated with arterial hypertension, hyperlipidemia, diabetes, smoking status, obesity, chronic lung disease and gender. On this occasion 17 miRs were identified to be significantly correlated with risk factors (Table 2), showing a correlation coefficient of at least >0.2 (both directions): miR-10b-3p, miR-10-5p, miR-17-3p, miR-21-5p, miR-151a-5p, miR-181a-5p, miR-185-5p, miR-194-5p, miR-199a-3p, miR-199b-3p, miR-212-3p, miR-363-3p, miR-548d-5p, miR-744-5p, miR-3117-3p, miR-5683 and miR-5701. 

**Figure 2:**
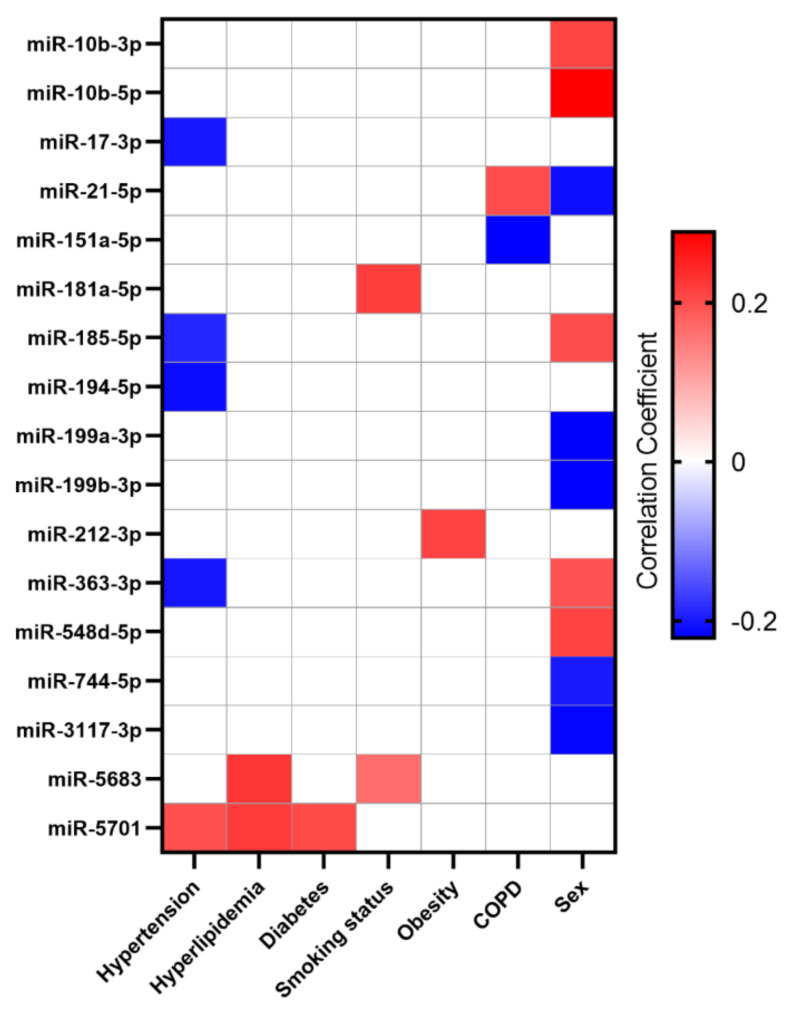
Heat-map of correlation coefficients between significant miRs and risk factors (arterial hypertension, hyperlipidemia, diabetes, smoking status, obesity, chronic lung disease and sex). Positive correlation is highlighted in red, negative correlation in blue. miRs with higher expression levels in female individuals compared to male individuals are illustrated in red and with lower expression in blue. The higher the correlation the brighter the color.

The heat-map illustrates positive and negative correlation coefficients between miRs and risk factors (Figure 2). 5 miRs were significantly correlated to at least 2 different risk factors. 7 miRs showed positive, 7 miRs showed negative, and 3 miRs showed positive and negative correlation to different risk factors. Most correlations between miRs and risk factors were found to be associated with gender (*n* = 10), followed by arterial hypertension (*n* = 5), hyperlipidemia (*n* = 2), smoking status (*n* = 2), chronic lung disease (*n* = 2), obesity (*n* = 1) and diabetes (*n* = 1). The strongest correlation was observed between gender and miR-10b-5p, with significantly higher expression values in women (correlation coefficient 0.29, *p* < 0.0001, FDR < 0.01). From a clinical perspective most interestingly–as risk factors can be targeted therapeutically–miR-5701 was positively correlated to arterial hypertension, hyperlipidemia and diabetes.

### 3.5. Clinical Relevance and Potential Confounders

In addition to correlations between miRs and cardiovascular risk factors, we also analyzed relevant clinical parameters and potential confounders. From a clinician’s perspective, clinical presentation is of critical interest. We identified two miRs that significantly correlated with acute non-ST-elevation myocardial infarction (NSTEMI). miR-629-5p was negatively correlated (correlation coefficient −0.20; *p* = 0.001; FDR 0.02) and miR-98-5p was positively correlated (0.21; *p* = 0.005; FDR 0.03) with NSTEMI. No significant association was found for EuroSCORE, severity of CAD as measured by the number of vessels affected, or inflammation as measured by circulating C-reactive protein (CRP) levels. Total cholesterol, LDL cholesterol, HDL cholesterol, and statin use were not significantly correlated with novel potential biomarker or treatment miRs from IMA tissue.

## 4. Discussion

**Figure 3:**
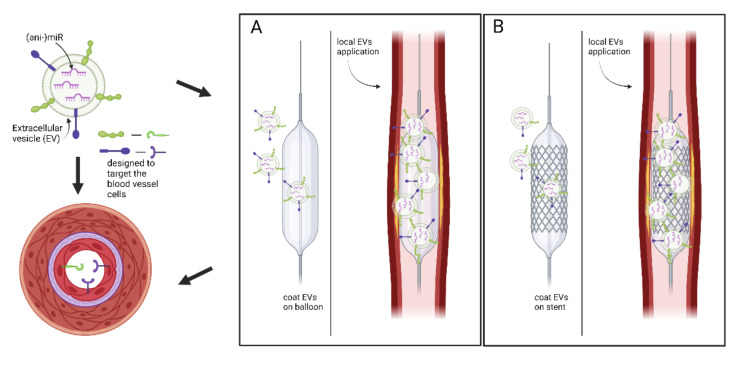
Potential therapeutic approaches based on miR perturbation in human coronary arteries. Coating of drug-eluting balloons (**A**) or stents (**B**) (similarly as proposed by Hu et al. [55]) with extracellular vesicles carrying miR, miR-mimics or anti-miRs has the potential to target specific cell types relevant for atherosclerosis in affected coronary arteries. Besides being inside EVs or conjugated to (lipo)proteins make extracellular miRNAs resistant to degradation.

This is the first study worldwide to investigate the correlation patterns between miR expressions and classical risk factors for CAD in a tissue-specific manner in IMA samples. Based on vascular tissue samples and comprehensive clinical information from 28 women and 160 men with angiographically confirmed CAD undergoing CABG surgery, we identified 17 novel miRs significantly correlated with cardiovascular risk factors. These miRs are promising candidates searching for new biomarkers and tissue-specific treatment approaches on the way to influence the course and therapy of cardiovascular diseases in a personalized manner. Additionally, miR-629-5p and miR-98-5p were identified to be significantly correlated with acute myocardial infarction (NSTEMI), highlighting these miRs as potential biomarkers or therapeutic targets in the setting of myocardial infarction treatment. Neither miR has been associated with acute myocardial infarction in the past. Figure 3 illustrates potential strategies to perturb local miR signatures based on percutaneous coronary intervention (PCI). Coated balloon or stent approaches with extracellular vesicles (EV)-carrying the ‘desired’ cargo, i.e., miR, miR-mimics or anti-miRs have the potential to deliver locally and cell type specific treatment solutions preventing proatherosclerotic effects. Besides, being inside EVs or conjugated to (lipo)proteins make extracellular miRs resistant to degradation [54]. 

### 4.1. miRNA Found to Play a Role in CAD/MI, Associations with Risk Factors 

Many miRs have been implicated as possible CAD biomarkers or effector molecules involved in underlying processes, as well as linked to known CAD risk factors [56,57]. Expressed in multiple tissues and cell types involved in atherosclerotic progression, for example endothelial cells or vascular smooth muscle cells, miRs have been found to have both pathogenic and protective effects [58]. A recent meta-analysis of 25 individual studies comparing miR expression in CAD patients to healthy controls, counted a total of 239 reported dysregulated miRs, however, after accounting for reproducibility only 48 of them were shown to be significantly dysregulated [59]. Several of these miRs have been previously associated with one or more traditional CAD risk factors, for example, arterial hypertension [60], hyperlipidemia [61,62,63,64], obesity [65,66], COPD [60,67] and diabetes [68,69]. These and other factors such as smoking and physical inactivity have been identified as typical risk factors by Framingham heart study and its follow-ups [70,71,72,73,74,75]. Synergistic effects between plasma miR-125b levels and classical risk factors age, sex, HDL cholesterol, fasting blood glucose and creatinine have been previously reported [28]. Lower expression of miR-145 in plasma has been significantly associated with diabetes and smoking and severity of CAD [76]. However, we did not identify these miRs in our study. Several miRs including miR-21 and mirR-181a have been previously associated with hypertension [77]. However, in our study, miR-21-5p was related to sex and COPD, whereas mirR-181a-5p was correlated with smoking status (Figure 2 and Table 2). miR-17 was down-regulated in chronic kidney disease patients with hypertension [78] and miR-17-3p demonstrates negative correlations with hypertension in our study. miR-151a-5p was down-regulated in plasma of COPD patients vs. smokers/non-smokers [79], whereas we identified it to be negatively correlated with COPD. However, all these previous findings were mainly related to blood plasma. To our knowledge, there was previously no comprehensive data available for miR signatures in vascular tissue. Our approach might stimulate further research on potential biomarkers, tissue and cell type specific treatment targets in human coronary arteries involving local miR signatures.

### 4.2. Potential Mechanisms of Selected Novel Mirnas Influencing Atherosclerosis Progression on Response to Risk Factors

For most of the newly identified miRs in IMA tissue, their potential mechanistic contribution to CAD is largely unknown. In the following section, we elucidate putative molecular mechanisms and functions of three selected miRs, identified in our study. 

**Figure 4:**
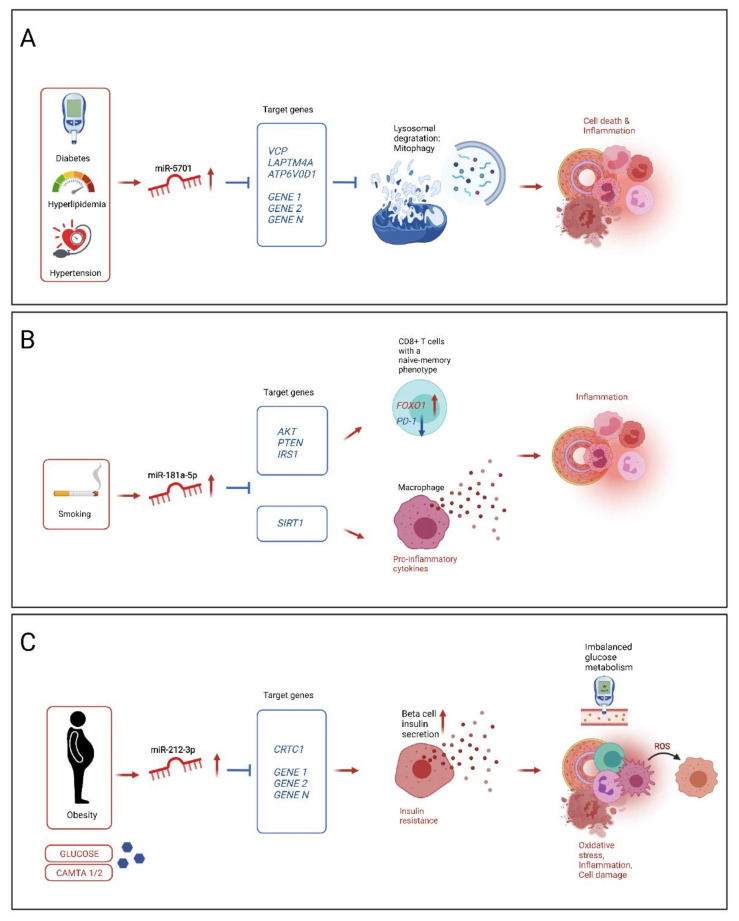
The proposed mechanism of action for (**A**) miR-5701. Hypertension, hyperlipidemia and/or diabetes lead to an increased expression of miR-5701 in IMA, which results in decreased expression of its putative target genes (e.g., VCP, LAPTM4A, ATP6V0D1) related to lysosomal biogenesis and mitochondrial quality control. This, on its turn, leads to impaired lysosomal degradation (mitophagy) and hence the accumulation of dysfunctional mitochondria, resulting in cell death and inflammation in the vascular tissue. (**B**) miR-181a-5p. Nicotine from cigarette smoke increases the expression of miR-181a-5p in CD8+ T cells. miR-181a-5p inhibits the expression of AKT, PTEN and IRS1, leading to increased FOXO1 activity and increased numbers of naïve-memory cells, which are known to express co-inhibitory receptor programmed death-1 (PD-1), acting as brakes on the immune system and inflammatory responses. Moreover, miR-181a-5p was identified to regulate the inflammatory response of macrophages in acute sepsis via SIRT1. (**C**) miR-212-3p. Obesity increases expression of miR-212-3p, via calmodulin binding transcription activators CAMTA1 and 2 and its target genes, stimulating insulin secretion from beta cells. CyclicAMP-Regulated Transcriptional Co-activator-1 (CRTC1) is targeted by miR-212, regulating insulin signaling and feed-back regulation of miR-212 and miR-132. Increased secretion of insulin results in insulin resistance promoting oxidative stress, inflammation and cell damage.

miR-5701 was observed to be positively correlated to arterial hypertension, hyperlipidemia and diabetes in our CAD patient cohort (Figure 2 and Table 2). It has been reported previously that miR-5701 expression was upregulated in patients with rheumatic aortic valve disease compared to individuals with calcified aortic valve disease [80]. In renal cell carcinoma miR-5701 promoted apoptosis by targeting phosphodiesterase-1B [81]. In Parkinson’s disease miR-5701 was reported to modulate mitochondrial-lysosomal cross talk, targeting genes involved in lysosomal biogenesis and mitochondrial quality control (VCP, LAPTMA4A, ATP6V0D1). MiR-5701 mimics induced mitochondrial dysfunction, defect in autophagy flux and cell death [82]. We therefore speculate that cell death/apoptosis and inflammation-related pathways are of primary relevance connecting miR-5701 and CAD, as proposed in Figure 4A.

In alignment with this, our previous investigations have demonstrated a massive down-regulation of mitochondrial genes, including the master regulators of mitochondrial biogenesis, coinciding with the time of most rapid atherosclerotic lesion expansion and foam cell formation in hypercholesterolemic mice [21].

Another candidate of interest is miR-181a-5p, which was positively correlated with active smoking status in our study (Figure 2 and Table 2). miR-181a-5p was reported to be upregulated in smokers before [83]. Nicotine exposure of CD8+ T-cells induced expression of miR-181a-5p in naïve-memory cells in vitro. miR-181a-5p inhibited expression of AKT, PTEN and IRS1 lead to increased FOXO1 activity and memory cell differentiation [83]. Expansion of memory CD8+ T-cells has been previously associated with acute coronary syndromes [84]. Individuals with increased fractions of CD8+ cells were characterized by decreased cytokine release from activated leukocytes, metabolic signs of insulin resistance and increased incidence of coronary events [85]. miR-181a-5p was identified to regulate the inflammatory response of macrophages in acute sepsis. Specific inhibitors significantly decreased the secretion of inflammatory factors in a mouse model [86]. Leukocytes are of specific interest in the development and progression of CAD. It can be assumed that active smoking increases miR-181a-5p expression and subsequently induces immune system activation and inflammation (Figure 4B).

miR-212-3p was positively correlated with obesity (Figure 2 and Table 2). Obesity, clinically evident as hyperglycemia or high blood glucose is known to increase the expression of miR-212-3p, possibly via calmodulin binding transcription activators CAMTA1 and 2 and its target genes, collectively increasing insulin secretion from beta cells in the pancreatic islets [87]. CyclicAMP-Regulated Transcriptional Co-activator-1 (CRTC1) is a known target of miR-212, participating in the regulation of insulin signaling and feed-back regulation of the miRNAs miR-212/miR-132 [88]. Increased secretion of insulin can result in insulin resistance, characterized by defects in glucose metabolism, triggering oxidative stress, inflammation and cell damage [89]. Calmodulin binding transcription activator 1 (CAMTA1) was reported to regulate miR-212-3p expression and insulin secretion. Exposure to glucose increased miR-212-3p expression in rat islets [87]. In a human cohort miR-212-3p was identified as potential biomarker for acute right heart failure with pulmonary artery hypertension [90]. Obesity is a chronic condition that can subsequently impact function of the right ventricle and pulmonary arterial pressure. Poor dietary habits could result in a perturbation of miR-212-3p expression with corresponding down-stream effects regarding imbalanced glucose metabolism, inflammation and cell death (Figure 4C).

In summary, increased expression of miR-5701, miR-181-5p and miR-212-3p in IMA samples were found to be positively correlated with treatable CV risk factors–namely arterial hypertension, hyperlipidemia, diabetes, smoking and obesity. This is of great clinical interest as local targeting of these miRs in coronary arteries might be a beneficial treatment strategy in the future. 

The conducted study incorporates several limitations. First, availability of healthy controls is not possible. Therefore, we are limited to cases only. Moreover, coronary artery tissue from CAD patients undergoing CABG are also not available. Hence, a potential substitute for coronary artery (and possibly the best alternative) represents IMA, which can be obtained during CABG surgery. Nevertheless, coronary arteries develop more pronounced atherosclerotic lesions compared to IMAs based on anatomic and functional differences. Second, detected correlations (showing correlation coefficients between 0.2–0.3) are rather weak, but reached good significance values (*p* < 0.01). We speculate that these patterns might increase in a larger cohort of CAD patients, preselected to cover specific risk factors of interest (e.g., heavy smokers vs. non-smokers). Third, the conducted study was not designed to identify underlying mechanisms or to further elucidate regulatory patterns by experimental validation. In particular, additional molecular layers of data such as transcriptome or proteome data of IMA in corresponding patients, in order to elucidate potential down-stream mechanisms of miRs or genome data to infer causality would be necessary. Fourth, assessed miRs in this study derive from IMA bulk tissue and do not allow to differentiate the impact of single miRs in specific cell types. Fifth, duration of statin use was not assessed and might explain weak correlations between cholesterol levels and identified miRs. Finally, the study cohort is limited to 192 individuals with underrepresentation of female individuals. Systematic comparisons of miR expression patterns in vascular tissue and body fluids in CAD or MI patients compared to healthy controls should be considered in well powered cohorts, to improve diagnostics and therapy in CAD and MI patients. In this respect, the provided miR atlas and our findings based on IMA samples might be a good starting point for a better elucidation of interactions between miRs, cardiovascular risk factors and CAD risk.

## 5. Conclusions

Our study is currently the largest CAD cohorts, providing miR profiles of IMA samples derived from individuals undergoing CABG surgery. Besides a comprehensive atlas (Table S1) extremely valuable for the field of cardiovascular research, this unbiased approach revealed 17 novel tissue specific miRs significantly correlated with cardiovascular risk factors. In addition, miR-629-5p and miR-98-5p appear to be of relevance in acute myocardial infarction. The results will stimulate further exploration of regulatory function of non-coding RNA, their relevance in CAD, and reveal tissue specific biomarkers and novel therapeutic targets. This is of tremendous clinical interest, as these miRs might be elegantly targeted via drug-eluting balloon or stenting strategies in a cell type specific manner in human coronary arteries (Figure 3).

## Figures and Tables

**Table 1:** Baseline characteristics of the study population categorized by gender. Continuous data are presented as mean ± standard deviation. The body mass index is the weight in kilograms divided by the square of the height in meters. The EuroSCORE indicates the percent risk of death within 30 days after surgery. The score is calculated with multivariable models that incorporate clinical predictors to estimate the operative mortality for any given patient.

Characteristics	Female (*n* = 28)	Male (*n* = 160)	*p*-Value
Age, years–mean ± SD	68.6 ± 7.0	70.6 ± 8.2	0.18
BMI *–mean ± SD	28.2 ± 6.1	28.2 ± 3.8	0.97
Rhythm–no. (%)			
Sinus rhythm at first presentation	26 (92.9)	127 (79.4)	0.12
Prior atrial fibrillation	5 (17.9)	34 (21.2)	0.80
CV * risk factors–no. (%)			
Arterial Hypertension	23 (82.1)	144 (90.0)	0.21
Hyperlipidemia	22 (78.6)	132 (82.5)	0.60
Diabetes mellitus	10 (35.7)	57 (35.6)	1.0
Use of insulin	2 (7.1)	18 (11.3)	0.74
Current smoker	9 (32.1)	27 (16.9)	0.07
Familial disposition	11 (39.3)	61 (38.1)	1.0
Obesity	9 (32.1)	63 (40.0)	0.53
Chronic renal disorder	1 (3.6)	19 (11.9)	0.32
Chronic lung disease	1 (3.6)	16 (10.0)	0.48
Clinical presentation–no. (%)			
Unstable angina	6 (21.4)	20 (12.5)	0.23
NSTEMI	8 (28.6)	38 (23.8)	0.63
Medical history–no. (%)			
Myocardial infarction	9 (32.1)	41 (25.6)	0.49
PCI *	7 (25.0)	42 (26.3)	1.0
CABG *	0 (0.0)	1 (0.6)	1.0
Stroke	2 (7.1)	14 (8.8)	1.0
Peripheral vascular disease	0 (0.0)	22 (13.8)	0.05
LV-EF * ≤50%–no./total no. (%)	4/21 (19.0)	33/131 (33.6)	0.22
Three vessel disease–no. (%)	19 (67.9)	143 (89.4)	0.006
EuroSCORE *–mean ± SD	6.46 ± 2.28	5.46 ± 3.13	0.05
Preoperative medication-no./total no. (%)			
Oral anticoagulant	6/20 (30.0)	35/120 (29.2)	1.0
Aspirin	19/22 (86.4)	121/138 (87.7)	0.74
P2Y_12_-inhibitor (Clopidogrel)	1/22 (4.5)	6/138 (4.3)	1.0
Betablocker	9/27 (33.3)	81/153 (52.9)	0.09
ACE-inhibitor^7^	11/27 (40.7)	71/153 (46.4)	0.68
Calcium channel inhibitor	4/20 (20.0)	29/117 (24.8)	0.78
Diuretic	7/27 (25.9)	56/153 (36.6)	0.38
Statin	22/28 (78.6)	126/160 (78.8)	1.0
Laboratory values at admission			
Hemoglobin, mg/dl	12.0 ± 3.5	14.0 ± 3.4	0.04
eGFR *, ml/min	73.0 ± 32.8	71.6 ± 24.0	0.82

* BMI: body mass index; CABG: coronary artery bypass grafting; CV: cardiovascular; eGFR: estimated Glomerular Filtration rate; EuroSCORE: European System for Cardiac Operative Risk Evaluation; LVEF: left-ventricular ejection fraction; NSTEMI: non-ST-elevation myocardial infarction; PCI: percutaneous coronary intervention.

**Table 2:** 17 miRs significantly correlated to at least one cardiovascular risk factor (clinical trait). Shown are correlation coefficients, adjusted *p*-values and false discovery rate (FDR). For miRs correlated to several clinical traits of interest correlation coefficients above 0.15 are provided.

miRNA	Clinical Trait	Correlation Coefficient	*p*-Value	FDR
miR-10b-3p	Sex	0.21	0.004	<0.05
miR-10b-5p	Sex	0.29	<0.001	<0.01
miR-17-3p	Hypertension	−0.20	0.006	<0.05
miR-21-5p	Sex	−0.21	0.004	<0.05
	COPD	0.20	0.005	<0.05
miR-151a-5p	COPD	−0.22	0.002	<0.05
miR-181a-5p	Smoking	0.22	0.003	<0.05
miR-185-5p	Sex	0.20	0.006	<0.05
	Hypertension	−0.19	0.009	0.06
miR-194-5p	Hypertension	−0.21	0.004	<0.05
miR-199a-3p	Sex	−0.22	0.002	<0.05
miR-199b-3p	Sex	−0.22	0.002	<0.05
miR-212-3p	Obesity	0.21	0.003	<0.05
miR-363-3p	Hypertension	−0.20	0.005	<0.05
	Sex	0.20	0.007	0.05
miR-548d-5p	Sex	0.21	0.004	<0.05
miR-744-5p	Sex	−0.20	0.006	<0.05
miR-3117-3p	Sex	−0.22	0.003	<0.05
miR-5683	Hyperlipidemia	0.23	0.002	<0.05
	Smoking	0.16	0.024	0.16
miR-5701	Hyperlipidemia	0.22	0.002	<0.05
	Diabetes	0.20	0.005	<0.05
	Hypertension	0.20	0.007	0.05

## Data Availability

All data used in this study are available in persistent repositories at the German Heart Centre Munich and can be requested from qualified researchers.

## Supplementary Materials

The following are available online at https://www.mdpi.com/article/10.3390/biom11111683/s1, Figure S1: miRNA quality assessment, Figure S2: miRNA adapter content before and after trimming, Table S1: miRNA atlas of vascular IMA tissue.
