# Supplementary material for: Vascular Tissue Specific miRNA Profiles Reveal Novel Correlations with Risk Factors in Coronary Artery Disease
Source: Biomolecules. 2021 Nov 12;11(11):1683. doi: 10.3390/biom11111683 (PMC8615466; doi:10.3390/biom11111683)
Supplement: Supplementary file 1 [file biomolecules-11-01683-s001.zip › biomolecules-1424782-supplementary/biomolecules-1424782 - supplementary.pdf]

biomolecules

MDPI

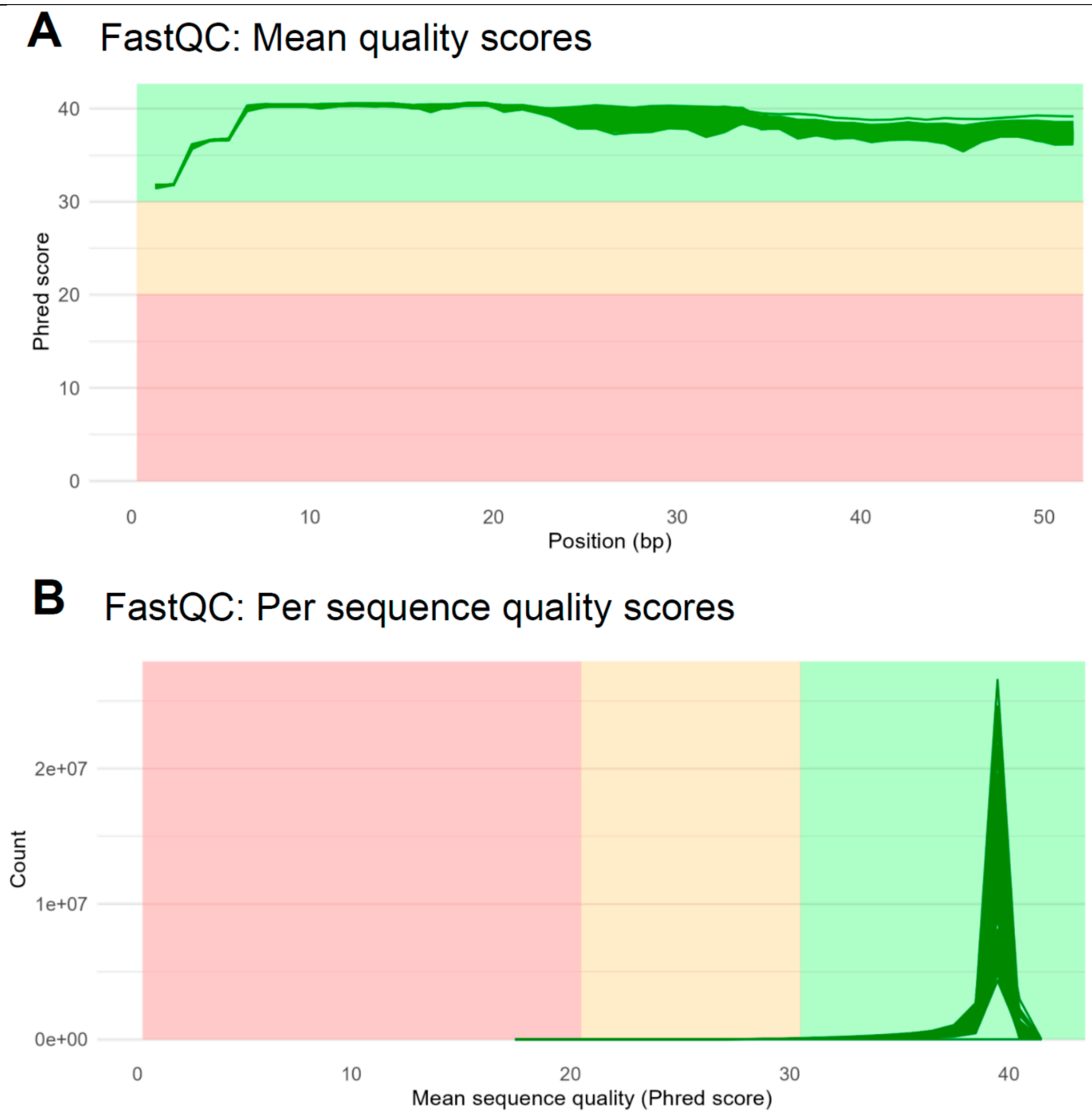

Figure S1. miRNA quality assessment using FastQC. Mean quality (phred) score per base (**A**) and sequence quality score distribution (**B**) for vascular IMA miRNA samples from CAD patients (*n*=191). Background colour indicates different quality thresholds – red:0–20 inacceptable, low sequence quality; yellow :20–30 acceptable, but not ideal; green: >30 excellent sequence quality.

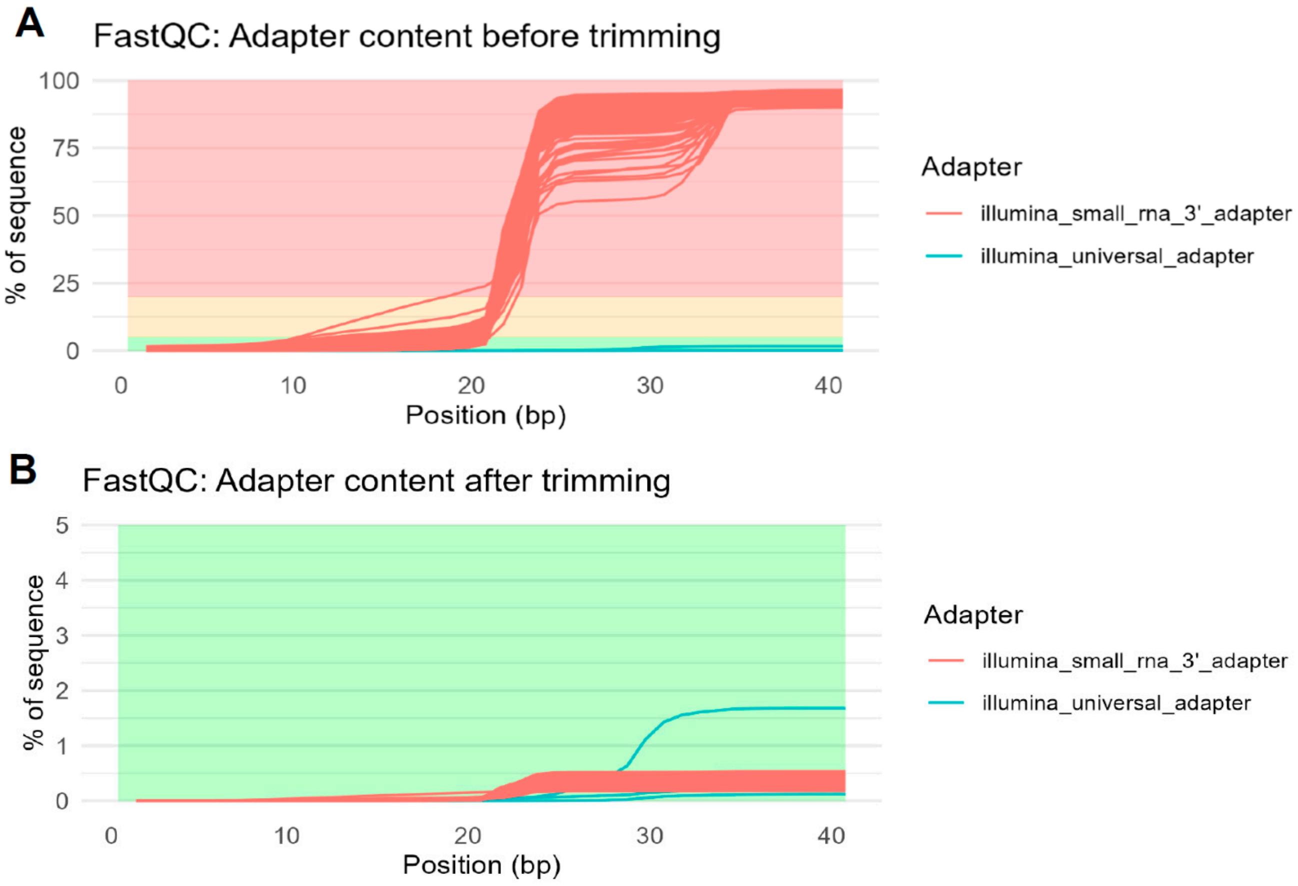

Figure S2. miRNA adapter content before and after trimming. Adapter content of vascular IMA miRNA sequences from CAD patients (n=191) before and aftert rimming. Background colour indicates accepted thresholds – green: 0 – 5% - excellent, minimal adapter content; yellow: 5 – 20% acceptable, but not ideal; red: 20 – 100% inacceptable high adapter content. As expected with miRNA data, unprocessed sequences show high adapter content after ~20 bp (**A**) which is the approximate length of target sRNAs. After performing adapter trimming with fastp, providing Illumina TruSeq Small RNA adapter sequence, adapter content dropped below 2% (**B**).
