# Supplementary material for: Vascular Tissue Specific miRNA Profiles Reveal Novel Correlations with Risk Factors in Coronary Artery Disease
Source: Biomolecules. 2021 Nov 12;11(11):1683. doi: 10.3390/biom11111683 (PMC8615466; doi:10.3390/biom11111683)
Supplement: Supplementary file 1 [file biomolecules-11-01683-s001.zip › biomolecules-1424782-supplementary/DigiMed Bayern Consortium_Co-authors.pdf]

*DigiMed Bayern Consortium:

Jonathan Adam, Institut für Medizinische Informationsverarbeitung Biometrie und Epidemiologie, Ludwig-Maximilians-Universität München, Munich, Germany; Institut für Epidemiologie, Helmholtz Zentrum München, Munich, Germany; **Reiner Anselm**, Institut Technik – Theologie – Naturwissenschaften, Ludwig-Maximilians-Universität München, Munich, Germany; **Sara Ates**, Deutsches Herzzentrum München, Klinik für Herz- und Kreislauferkrankungen, Technische Universität München, Munich, Germany; **Sabine Bauer**, Deutsches Herzzentrum München, Klinik für Herz- und Kreislauferkrankungen, Technische Universität München, Munich, Germany; **Nicole Beck**, Deutsches Herzzentrum München, Klinik für Herz- und Gefäßchirurgie, Technische Universität München, Munich, Germany; **Jürgen Beckmann**, Fakultät für Sport- und Gesundheitswissenschaften, Technische Universität München, Munich, Germany; **Riccardo Berutti**, Institut für Neurogenomik, Helmholtz Zentrum München, Munich, Germany; **Stefan Brandmaier**, Institut für Epidemiologie, Helmholtz Zentrum München, Munich, Germany; **Theresa Brunet**, Institut für Humangenetik, Technische Universität München, Munich, Germany; **Salvatore Cassese**, Deutsches Herzzentrum München, Klinik für Herz- und Kreislauferkrankungen, Technische Universität München, Munich, Germany; **Manuela Decker**, Deutsches Herzzentrum München, Klinik für Herz- und Kreislauferkrankungen, Technische Universität München, Munich, Germany; **Martin Dichgans**, Institut für Schlaganfall- und Demenzforschung, Ludwig-Maximilians-Universität München, Munich, Germany; **Philine Diesselhorst**, Institut Technik – Theologie – Naturwissenschaften, Ludwig-Maximilians-Universität; **Horst Domdey**, BioM Biotech Cluster Development GmbH, Martinsried, Germany; **Martina Dreßen**, Deutsches Herzzentrum München, Klinik für Herz- und Gefäßchirurgie, Technische Universität München, Munich, Germany; **Arne Dressler**, Institut Technik – Theologie – Naturwissenschaften, Ludwig-Maximilians-Universität München, Munich, Germany; **Florent Dufour**, Leibniz-Rechenzentrum, Munich, Germany; **Sven Duscha**, Deutsches Herzzentrum München, Klinik für Herz- und Kreislauferkrankungen, Technische Universität München, Munich, Germany; **Hans-Henning Eckstein**, Klinik und Poliklinik für Vaskuläre und Endovaskuläre Chirurgie, Klinikums rechts der Isar, Technische Universität München, Munich,

Germany; **Aiman Farzeen**, Institut für Humangenetik, Technische Universität München, Munich, Germany; Institut für Epidemiologie, Helmholtz Zentrum München, Munich, Germany; **Therese Feiler**, Institut Technik – Theologie – Naturwissenschaften, Ludwig-Maximilians-Universität München, Munich, Germany; **Ines Gall**, Deutsches Herzzentrum München, Institut für Laboratoriumsmedizin, Technische Universität München, Munich, Germany; **Ulrich M. Gassner**, Juristische Fakultät, Universität Augsburg, Augsburg, Germany; **Christian Gieger**, Institut für Epidemiologie, Helmholtz Zentrum München, Munich, Germany; **Monica Gotor-Blazquez**, Deutsches Herzzentrum München, Klinik für Herz- und Kreislauferkrankungen, Technische Universität München, Munich, Germany; **Ulrich Güldener**, Deutsches Herzzentrum München, Klinik für Herz- und Kreislauferkrankungen, Technische Universität München, Munich, Germany; **Nicolay Hammer**, Max-Planck-Institut für Biochemie, Munich, Germany; **Johann Hawe**, Deutsches Herzzentrum München, Klinik für Herz- und Kreislauferkrankungen, Technische Universität München, Munich, Germany; **Thomas Hendel**, Institut für Epidemiologie, Helmholtz Zentrum München, Munich, Germany; **Stefan Holdenrieder**; Deutsches Herzzentrum München, Institut für Laboratoriumsmedizin, Technische Universität München, Munich, Germany; **Stephan Jonas**, Institut für Informatik, Technische Universität München, Munich, Germany; **Adnan Kastrati**, Deutsches Herzzentrum München, Klinik für Herz- und Kreislauferkrankungen, Technische Universität München, Munich, Germany; **Wolfgang Kempf**, Klinikums rechts der Isar, Technische Universität München, Munich, Germany; **Thorsten Keßler**, Deutsches Herzzentrum München, Klinik für Herz- und Kreislauferkrankungen, Technische Universität München, Munich, Germany; **Wolfgang Koenig**, Deutsches Herzzentrum München, Klinik für Herz- und Kreislauferkrankungen, Technische Universität München, Munich, Germany; **Florian Kohlmayer**, Bitcare, Munich, Germany; **Markus Krane**, Deutsches Herzzentrum München, Klinik für Herz- und Gefäßchirurgie, Technische Universität München, Munich, Germany; **Dieter Kranzlmüller**, Institut für Informatik, Ludwig-Maximilians-Universität München, Munich, Germany; **Harald Lahm**, Deutsches Herzzentrum München, Klinik für Herz- und Gefäßchirurgie, Technische Universität München, Munich, Germany; **Rüdiger Lange**, Deutsches Herzzentrum München, Klinik für Herz- und Gefäßchirurgie, Technische Universität München,

Munich, Germany; **Andreas Lehmann**, Bitcare GmbH, Munich, Germany; **Ling Li**, Deutsches Herzzentrum München, Klinik für Herz- und Kreislauferkrankungen, Technische Universität München, Munich, Germany; **Birgit Linkohr**, Institut für Epidemiologie, Helmholtz Zentrum München, Munich, Germany; **Lars Maegdefessel**, Klinik und Poliklinik für Vaskuläre und Endovaskuläre Chirurgie, Klinikums rechts der Isar, Technische Universität München, Munich, Germany; **Matthias Mann**, Max-Planck-Institut für Biochemie, Munich, Germany; **Rainer Malik**, Institut für Schlaganfall- und Demenzforschung, Ludwig-Maximilians-Universität München, Munich, Germany; **Thomas Meitinger**, Institut für Humangenetik, Technische Universität München, Munich, Germany; **Irina Neb**, Deutsches Herzzentrum München, Klinik für Herz- und Gefäßchirurgie, Technische Universität München, Munich, Germany; **Tina O'Hehir**, Deutsches Herzzentrum München, Klinik für Herz- und Kreislauferkrankungen, Technische Universität München, Munich, Germany; **Shichao Pang**, Deutsches Herzzentrum München, Klinik für Herz- und Kreislauferkrankungen, Technische Universität München, Munich, Germany; **Benedikt Perl**, Fakultät für Sport- und Gesundheitswissenschaften, Technische Universität München, Munich, Germany; **Annette Peters**, Institut für Medizinische Informationsverarbeitung Biometrie und Epidemiologie Ludwig-Maximilians-Universität München, Munich, Germany; Institut für Epidemiologie, Helmholtz Zentrum München, Munich, Germany; **Fatemeh Peymani**, Institut für Humangenetik, Technische Universität München, Munich, Germany; **Roland Pichler**, Max-Planck-Institut für Biochemie, Munich, Germany; **Heiko Pfister**, Deutsches Herzzentrum München, Institut für Laboratoriumsmedizin, Technische Universität München, Munich, Germany; **Paola Pisano**, Max-Planck-Institut für Biochemie, Munich, Germany; **Holger Prokisch**, Institut für Humangenetik, Technische Universität München, Munich, Germany; **Lara Marie Reimer**, Institut für Informatik, Technische Universität München, Munich, Germany; **Michaela Sander**, Deutsches Herzzentrum München, Institut für Laboratoriumsmedizin, Technische Universität München, Munich, Germany; **Veronika Sanin**, Deutsches Herzzentrum München, Klinik für Herz- und Kreislauferkrankungen, Technische Universität München, Munich, Germany; **Lea Dewi Schlieben**, Institut für Humangenetik, Technische Universität München, Munich, Germany; **Yannick Schlote**, Institut Technik – Theologie – Naturwissenschaften,

Ludwig-Maximilians-Universität München, Munich, Germany; **Sofie Schmid**, Klinikums rechts der Isar, Technische Universität München, Munich, Germany; **Raphael Schmieder**, Deutsches Herzzentrum München, Klinik für Herz- und Kreislauferkrankungen, Technische Universität München, Munich, Germany; **Heribert Schunkert**, Deutsches Herzzentrum München, Klinik für Herz- und Kreislauferkrankungen, Technische Universität München, Munich, Germany; **Megi Sharikadze**, Leibniz-Rechenzentrum, Munich, Germany; **Ankit Sinha**, Max-Planck-Institut für Biochemie, Munich, Germany; Fabian Starnecker, Deutsches Herzzentrum München, Klinik für Herz- und Kreislauferkrankungen, Technische Universität München, Munich, Germany; **Medini Steger**, Max-Planck-Institut für Biochemie, Munich, Germany; **Sophia Steigerwald**, Max-Planck-Institut für Biochemie, Munich, Germany; Ruoyu Sun, BioM Biotech Cluster Development GmbH, Martinsried, Germany; **Moritz von Scheidt**, Deutsches Herzzentrum München, Klinik für Herz- und Kreislauferkrankungen, Technische Universität München, Munich, Germany; **Matias Wagner**, Institut für Humangenetik, Technische Universität München, Munich, Germany; **Annie Westerlund**, Deutsches Herzzentrum München, Klinik für Herz- und Kreislauferkrankungen, Technische Universität München, Munich, Germany; **Jens Wiehler**, BioM Biotech Cluster Development GmbH, Martinsried, Germany; **Michael Wierer**, Max-Planck-Institut für Biochemie, Munich, Germany; **Peter Zinterhof**, Leibniz-Rechenzentrum, Munich, Germany;
